# Antipredator behavior of newts (*Cynops pyrrhogaster*) against snakes

**DOI:** 10.1371/journal.pone.0258218

**Published:** 2021-11-29

**Authors:** Koji Mochida, Akira Mori

**Affiliations:** 1 Department of Zoology, Graduate School of Science, Kyoto University, Kyoto, Japan; 2 Wildlife Research Center, Kyoto University, Kyoto, Japan; 3 Primate Research Institute, Kyoto University, Kyoto, Japan; 4 Nagasaki Institute of Applied Science, Kyoto, Japan; University of Regina, CANADA

## Abstract

Newts and salamanders show remarkable diversity in antipredator behavior, developed to enhance their chemical defenses and/or aposematism. The present study reports on the antipredator behavior of newts (*Cynops pyrrhogaster*) in response to snakes. Newts displayed a significant amount of tail-wagging and tail-undulation in response to a contact stimulus from the snake’s tongue, which is a snake-specific predator stimulus, as compared to a control stimulus (behavioral scores: tongue, 1.05 ± 0.41; control, 0.15 ± 0.15). Newts that were kept in warm temperature conditions, 20°C (at which snakes are active in nature), performed tail displays more frequently than newts kept in low-temperature conditions, 4°C (at which snakes are inactive in nature). Our results suggest that the tail displays of *C*. *pyrrhogaster* could function as an antipredator defense; they direct a snake’s attention to its tail to prevent the snake from attacking more vulnerable body parts. We also discussed the reason for inter-populational variation in the tendency of newts to perform tail displays.

## Introduction

Newts and salamanders have evolved remarkably diverse secondary defensive traits to avoid predation [1]. Brodie [2] reported the convergent evolution of antipredator behaviors among Salamandrid, Ambystomatid, Hynobiid, and Plethodontid species. These behaviors correlated with the distribution of granular glands that secrete various types of toxic and noxious chemicals, the types of chemicals, and/or their aposematism. In one species of Salamandridae (*Cynops pyrrhogaster*), antipredator behaviors vary between populations, depending on the protective effect of a specific behaviors against their local predators [3,4]. Newts and salamanders from Salamandridae and Plethodontidae can also change their antipredator behavior with changes in ambient temperature owing to the dependence of their running ability on their body temperature [5–7] and the activity patterns of their predators at different ambient temperatures [8]. Therefore, both biotic and abiotic environmental factors shape the antipredator behavior of newts and salamanders.

Newts (*Cynops pyrrhogaster*) release tetrodotoxin, a neurotoxin that blocks sodium channels present in most vertebrates, from the skin glands present on their dorsal surface and tail [9]. Furthermore, newts exhibit immobile antipredator behavior known as the unken reflex, a defensive posture which displays the aposematic red coloration of their venter [1,3,10]. This behavior enhances the effectiveness of aposematism, which prevents attacks from predators that hunt visually, such as birds, whose tetrachromatic vision enables them to distinguish red coloration [11]. Therefore, *C*. *pyrrhogaster* found on islands, whose main potential predators are birds, use physiological resources such as carotenoids to perform the unken reflex at a high frequency [4,12]. However, this immobile aposematic behavior may result in death for the newts when performed for non-color oriented hunters, such as mammalian carnivores, because it precludes their opportunity for escape. Therefore, newts found on the mainland, whose main potential predators include both birds and mammalian carnivores, do not display this behavior as frequently and have evolved a higher level of toxicity that affects both predators [12].

Newts and salamanders also exhibit tail displays that are effective against snakes and small mammals [2], which are generally not color-oriented hunters, although some of them have trichromatic vision [13–15]. The tail-lashing display can directly repel these predators when granular glands are concentrated on the tail [16]. Tail-wagging and tail-undulation direct the predator’s attention to the tail which prevents an attack on other body parts [2].

*Cynops pyrrhogaster* is exposed to predation pressures from birds, mammalian carnivores, and snakes [3,17,18]. However, predator–prey interactions between *C*. *pyrrhogaster* and snakes have not been well-studied, especially from a behavioral perspective. We conducted behavioral experiments in a laboratory to determine whether *C*. *pyrrhogaster* displays antipredator behaviors using the tail, especially in response to snake-specific predator stimuli. We also conducted the experiment under various temperature conditions, which affect the activity patterns of snakes in the wild. Finally, we preliminarily compared the tail-display tendencies of newt populations with different local biota. We predicted that newts on the mainland that are exposed to predation pressures from mammalian carnivores would not use the tail display; similar to the unken reflex, newts might lose the opportunity to escape if they perform the display.

## Materials and methods

### Ethics

All procedures complied with the required regulations of Kyoto university for animal experimentation of Kyoto University. The study was approved by the Committee on the Ethics of Animal Experiments of the Kyoto University.

### Animal sampling

**Fig 1:**
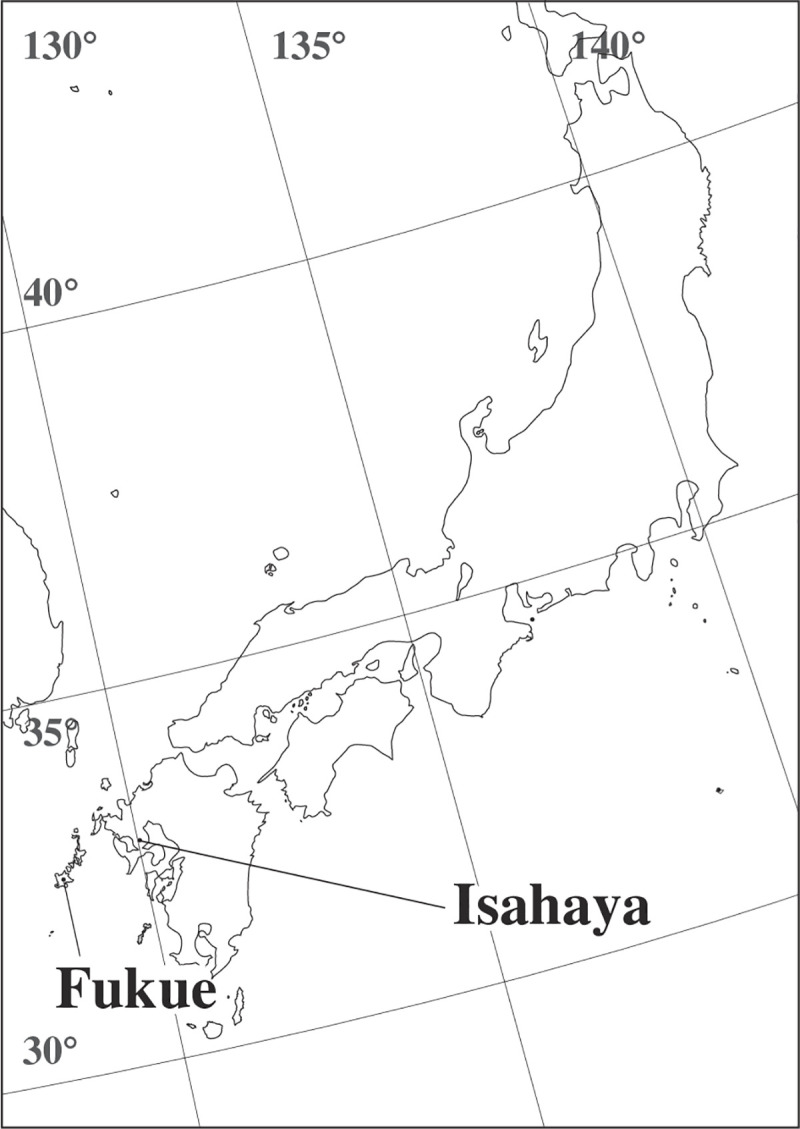
Map showing the sampling sites. We collected newts (*Cynops pyrrhogaster*) from Fukue Island (Fukue) and the mainland Japan (Isahaya).

We collected newts (*C*. *pyrrhogaster*) from Fukue Island (Fukue, Nagasaki Prefecture, Japan; N 32° 42’ and E 128° 46’) and the mainland Japan (Isahaya, Nagasaki Prefecture, Japan; N 32° 52’ and E 130° 1’) (Fig 1). There were no restrictions on newt collection at either field site. There was no difference in the presence of bird and snake predator species between the island or mainland locations of newt populations, including Fukue and Isahaya populations [3]. However, there were fewer mammalian species present on the islands, especially Fukue Island, than the mainland; newts on Fukue Island had the least amount of exposure to predation pressures from mammalian carnivores [4]. In 2004, we collected 39 individual newts by hands from Fukue Island (for Experiments 1, 2, and 3). In the same year, we collected 17 individuals by hands from the mainland at Isahaya (for Experiment 3). In both locations, we observed a potential snake predator (*Gloydius blomhoffii*) while collecting the newts.

For the experiments, newts were placed in individual cages (150 mm × 150 mm × 50 mm) of which substrate was damp paper towels. All experiments were conducted in the laboratory; the temperature was maintained at 20°C and the light/dark cycle was set at 12h/12h during the experimental period. Newts were held in these conditions for approximately 1 week following their capture. We released the newts back to their respective sampling sites after the all experiments were conducted.

### Experiment 1

To examine whether the newts performed tail displays in response to snakes, we used 20 individual newts obtained from an insular population (Fukue), which were maintained in the laboratory at 20°C for one day prior to the experiment. We also used three laboratory-reared individual colubrid snakes (*Elaphe quadrivirgata*). An experimenter gently held one of the snakes by its neck and allowed it to touch the head of a newt with its tongue. The tongue stimulus is a snake-specific predator stimulus that elicits antipredator behavior in newts and salamanders [19]. As a control stimulus, we used snakes whose mouths were sealed with adhesive tape and an experimenter allowed the snake to touch the head of the newt with only its snout. In the first trial, we presented the tongue stimulus to half (10) of the newts. The stimulus was presented five times at 1-min intervals for each newt. In the second trial, the next day, we presented the control stimulus five times at 1-min intervals to the same newt. For the remaining 10 individuals, we presented the control stimulus in the first trial and tongue stimulus in the second trial. For each stimulus, we recorded whether the newts performed any of the following three tail displays (using 0 or 1): “tail-lashing” (the tail forcibly attacks the predator), “tail-wagging” (the tail becomes upright and held straight, swinging from side to side), and “tail-undulation” (the tail moves in a sinuous manner). We calculated the number of behavioral responses based on a scoring system (0–5), with a score of 0 indicating that a newt did not perform any tail displays in response to any of the five stimuli in a trial and with a sore of 5 indicating that a newt performed the tail displays in response to all five stimuli in a trial. We compared the tail display scores between stimuli (snake’s tongue or snout) with the Wilcoxon signed rank test.

### Experiment 2

We examined the effect of temperature on the performance of tail displays in newts using warm conditions (20°C), at which they would encounter snakes in the wild, and under low-temperature conditions (4°C), at which the snakes are inactive. Newts are also less active (but not inactive) at 4°C both in the laboratory [7] and the wild (Mochida, personal observations). We used 19 individuals from an insular population (Fukue) for this experiment. We maintained the newts either in the laboratory (20°C) or in a refrigerated chamber (4°C) for one day prior to the experiment. In the first trial, using the blunt end of forceps as a general predator stimulus, the experimenter gently picked the head of a newt one time. In the second trial, the next day, we exerted the same predator stimulus one time on the same newt. Ten of the individuals received the predator stimulus at 4°C in the first trial and at 20°C in the second trial; the remaining nine received the predator stimulus at 20°C in the first trial and at 4°C in the second trial. We recorded whether a newt performed a tail display (tail-lashing, tail-wagging, or tail-undulation), the unken reflex, or no display at all (this category includes the escape response). We compared the frequency of antipredator behavior (tail displays, unken reflex, or no display) between experimental temperatures (4 and 20°C) with χ^2^ test.

### Experiment 3

We compared the tendency of newts to perform tail displays between populations with different types of predators present (insular vs. mainland populations). We presented the snake tongue as a contact stimulus to newts from the mainland population (Isahaya) using the same method as in Experiment 1. We recorded and scored the tendency of newts to perform tail displays (tail-lashing, tail-wagging, or tail-undulation) in response to five predator stimuli per trial. We compared the tail display score, induced by a snake’s tongue at 20°C, between island (results of Experiment 1) and mainland populations with the Wilcoxon rank sum test.

#### Software for statistics

R version 3.5.0 with the package “exactRankTests” was used for the statistical analyses (R Core Development Team).

## Results

**Fig 2:**
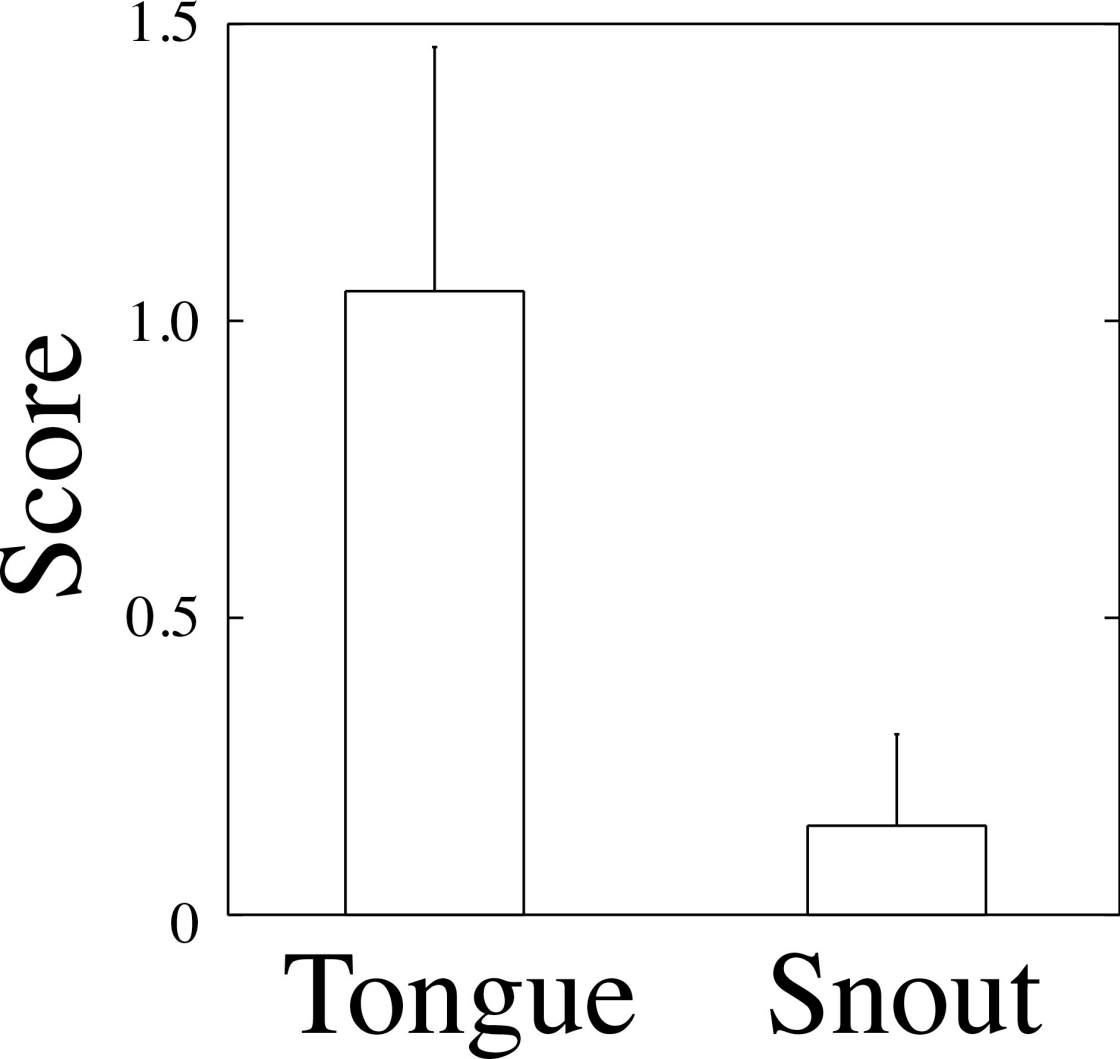
Newts (*C*. *pyrrhogaster*) displayed tail-wagging and tail-undulation in response to snake-specific predator stimuli. Newts performed tail displays at a higher frequency in response to contact with snake tongues than with snake snouts (Wilcoxon signed rank test, *V* = 21, *P* = 0.031). Error bars indicate SE.

Newts from the insular population (Fukue) performed tail displays more frequently in response to the contact stimulus of a snake tongue than a snake snout (scores: tongue, 1.05 ± 0.41; snout, 0.15 ± 0.15) (Wilcoxon signed rank test, *V* = 21, *P* = 0.031) (Fig 2). The tail displays performed by 6 of the 20 individuals were as follows: tail-wagging (where the tail is upright and raised away from the substrate, swinging from side to side) and tail-undulation (where the tail moves sinuously). No stimuli induced tail-lashing behavior (where the tail strikes the snakes). Newts that did not perform any tail display in response to predator stimuli engaged in locomotary escape.

**Fig 3:**
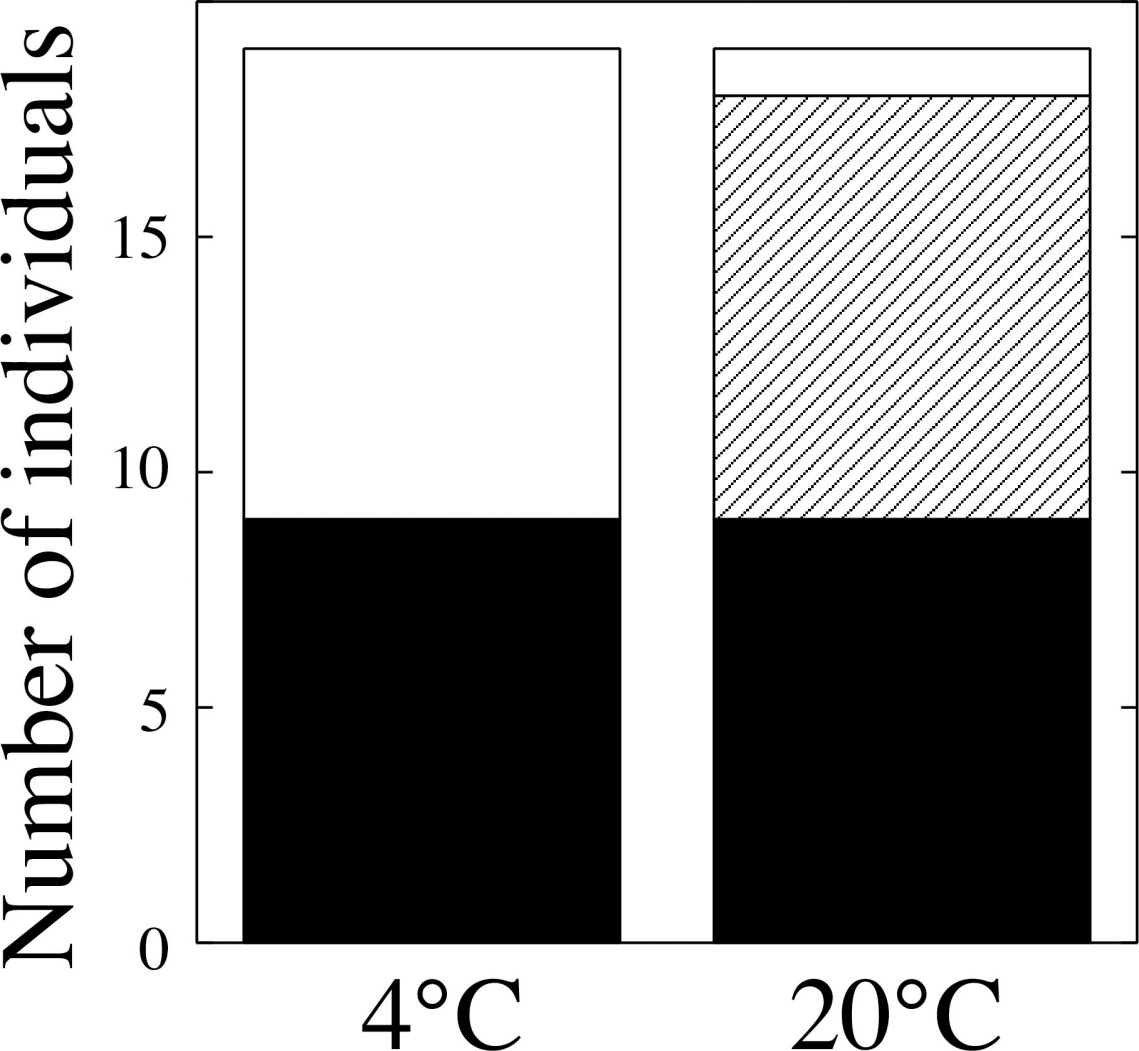
Newts (*C*. *pyrrhogaster*) performed tail displays in response to predator stimuli in warm temperature (20°C) and low-temperature (4°C) conditions. White, striped, and black columns indicate the number of individuals performing the unken reflex, tail displays, and no display (including escape response), respectively. Error bars indicate SE.

Tail-wagging and tail-undulation were displayed by 9 of the 20 newts that were kept in warm conditions (20°C), but not by any newts kept in low-temperature conditions (4°C) (Fig 3). Half (10) of the newts kept in low-temperature conditions performed the unken reflex and remaining individuals (9) performed locomotary escape in response to the predator stimulus (forceps), but their speed of escape was slower at 4°C than at 20°C. Thus, newts at different temperatures exhibited distinctly different antipredator behaviors (χ^2^ test, χ^2^ = 16.364, df = 2, *P* < 0.001).

**Fig 4:**
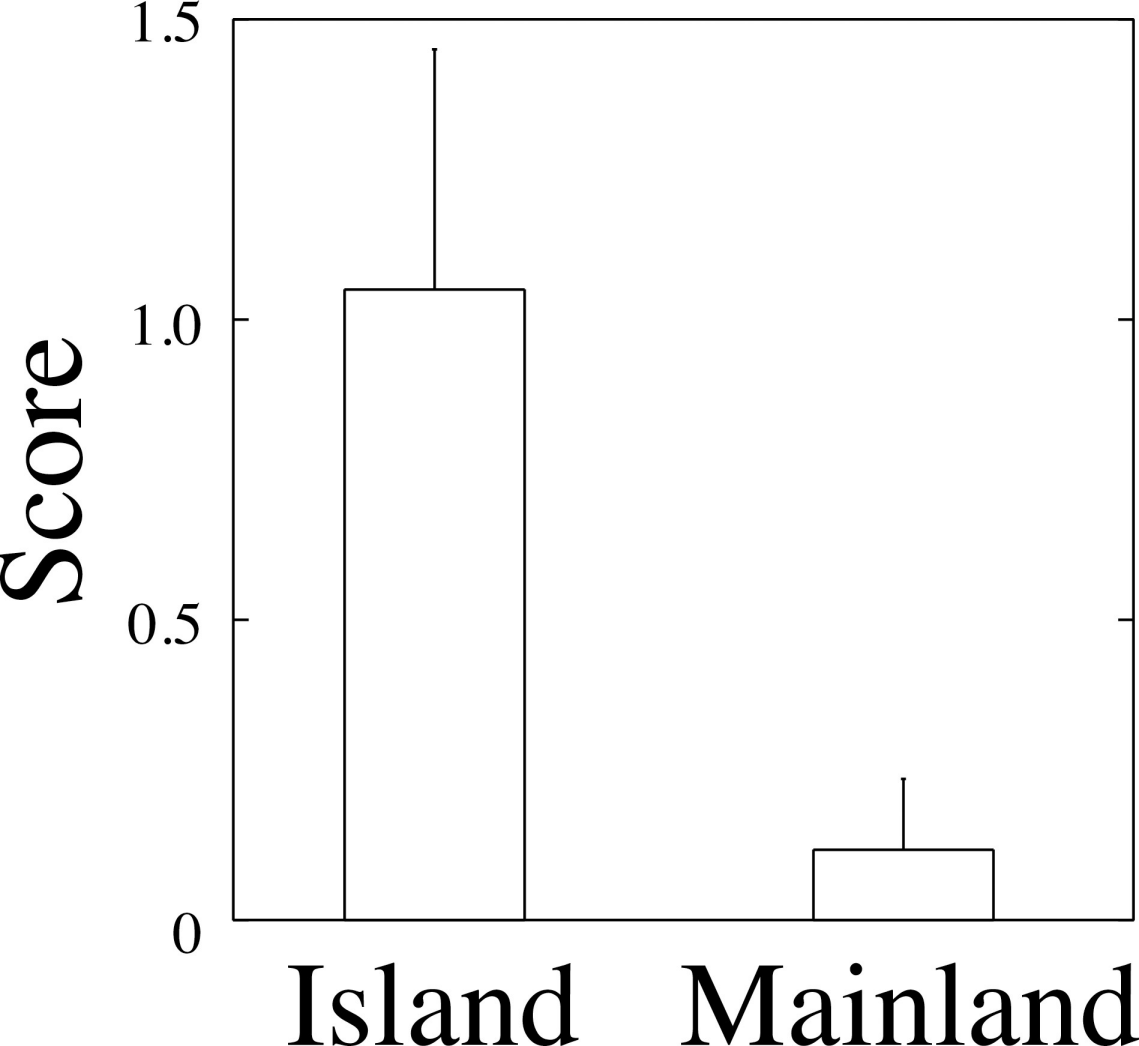
Newts (*C*. *pyrrhogaster*) from an island population displayed more tail-wagging and tail-undulation than those from the mainland population, but the difference was not significant (Wilcoxon rank sum test, *W* = 213, *P* = 0.052). Error bars indicate SE.

Newts from the insular population (Fukue) performed tail displays more frequently than those from the mainland population (Isahaya) (scores: insular, 1.05 ± 0.41; mainland, 0.12 ± 0.12), although the difference fell short of the significance level (Wilcoxon rank sum test, *W* = 213, *P* = 0.052) (Fig 4). From the mainland population, 1 out of 17 individuals performed tail displays.

## Discussion

Newts performed tail-wagging and tail-undulation in response to a snake-specific predator stimulus (tongue) and a general predator stimulus (forceps). Tail-wagging and tail-undulation function as an antipredator defense that directs a predator’s attention to the tail which contains toxic and noxious glands [1,2]. Granular glands are distributed on the dorsal surface and tail that secrete tetrodotoxin, a neurotoxin in vertebrates [9]. Upon injury, this species can regenerate its tail [20]. A newt tail can be unprofitable for predators (such as birds, mammals, and snakes) and it would not be a crucial loss for newts, due to their regenerative ability [20]. However, a predator attacking a newt’s head can prove fatal, even if the predator does not consume the rest of the newt to avoid the granular glands on its dorsal surface (Mochida, personal observation). As opposed to a previous study [16], no tail-lashing was observed at all. The distribution of toxic glands and the regenerative ability of *C*. *pyrrohogaster* support the hypothesis that tail displays function as an antipredator defense that directs predator’s attention to the expendable tail and prevents attacks on other body parts, such as the head.

Tail displays were induced by the snake-specific contact stimulus of the tongue, but not the snout. Like snakes, several lizard species detect and identify foods by flicking a tongue before attacking [21], but no lizards are known to prey on amphibians in Japan. Newts performed tail displays at warm temperatures, at which snakes are active in the wild [22]. In contrast to snakes, avian and mammalian predators are active throughout the winters, and a temperature of 4°C has been recorded at the sampling sites [3]. In the wild, newts are less active, but not inactive, at 4°C (Mochida, personal observations). In our study, at low temperatures (4°C), newts did not perform tail displays, but rather the unken reflex, which functions to avert avian predators [11]. We did not test whether the tail displays function to defend against avian or mammalian predators. However, based on the type of contact stimulus and ambient temperature at which tail displays were strongly exhibited, we presumed that the main targets would be snakes.

Newts on the mainland did not exhibit antipredator tail displays in response to a snake-specific predator stimulus (Fig 3) nor a general predator stimulus (forceps) [7]. We predicted that newts on the mainland, which are exposed to predation pressures from mammalian carnivores, would not perform the tail display; these newts would most likely devote their time for locomotary escape, due to the low effectiveness of static displays against mammals. Our results partially support this prediction, although we only compared newts from one island and one mainland population. The inter-populational variation in the frequency of tail displays detected in our study may be due to different degrees of predation pressure from snakes (*Gloydius blomhoffii*) on newts between islands and the mainland. While the contribution of tail displays towards newt survival rates and the variation in their frequency requires further research, the results of our study suggest that the tail displays of *C*. *pyrrhogaster* function as an antipredator defense that directs a snake’s attention to that tail and prevents attacks on more vulnerable body parts.

## Supporting information

S1 File (XLSX)
